# Complement Activity in the Egg Cytosol of Zebrafish *Danio rerio*: Evidence for the Defense Role of Maternal Complement Components

**DOI:** 10.1371/journal.pone.0001463

**Published:** 2008-01-23

**Authors:** Zhiping Wang, Shicui Zhang, Guangfeng Wang, Yan An

**Affiliations:** Department of Marine Biology, Ocean University of China, Qingdao, People's Republic of China; National Institute on Aging, United States of America

## Abstract

Most fish embryos that develop externally are exposed to an environment full of microbes. How they survive microbial attacks are not understood to date. Here we demonstrated that the egg cytosol prepared from the newly fertilized eggs of zebrafish *Danio rerio* is capable of killing the Gram-negative bacterium *Escherichia coli*, via *in vitro* assay system of the complement activity established. All findings indicate that it is the complement system operating via the alternative pathway that is attributable to the bacteriolytic activity. This is the first report providing the evidence for the functional role of the maternal complement components in fish eggs, paving the way for study of maternal immunity in other organisms whose eggs are fertilized *in vitro*.

## Introduction

Most fish embryos that develop externally are exposed to an environment full of microbes, many of which are pathogens capable of killing other organisms. Furthermore, maturation of immunocompetence in fish develops quite late although both lymphoid organs and T- and B-lymphocytes may appear early in embryogenesis [1]. Therefore, developing fish embryos and larvae have little or only limited ability to synthesize immune-related molecules endogenously [2], [3]. How developing fish embryos and larvae survive microbial attacks is one of the central problems for reproductive and developmental immunology, but little information as such is available to date.

Complement system consisting of approximately 35 plasma and membrane-bound proteins comprises one of the first lines of defense against pathogenic infection by alerting host the presence of potential pathogens as well as clearing pathogens. There are three pathways by which the complement system can be activated: the classical pathway (CP), the alternative pathway (AP) and the lectin pathway (LP). The CP activation is initiated by binding of antibody to the C1 complex, formed by C1q and two serine proteases (C1r and C1s), or by direct binding of the C1q component to the pathogen surface, and requires both Ca^2+^ and Mg^2+^ [4]–[6]. The AP is mainly triggered by the certain structures on microbial surface in an antibody-independent manner, and requires Mg^2+^ alone [7], [8]. The C3 is cleaved spontaneously in plasma to yield C3b which interacts non-covalently with factor B (Bf) and factor D, resulting in the formation of the alternative C3 convertase [9]. The LP is activated by binding of microbial polysaccharides to circulating lectins, such as mannose-binding lectin (MBL), and requires Ca^2+^ [10]–[13]. MBL binds to mannose residues which then results in the cleavage of C4 via mannose-binding protein-associated serine esterase. All the three pathways merge at a common amplification step involving C3, a central complement component being a part of all the three pathways, and proceed through a terminal pathway that leads to the formation of a membrane attack complex, which can directly lyse microbial cells. Maternal transfer of complement components such as C3, C4, C5 and Bf to offspring has recently been demonstrated in rainbow trout [14]. Maternal transmission of C3 has also been reported in carp [15] and spotted wollfish [16]. Likewise, immunoglobulins (IgM) has been shown to be present in eggs and early embryos of several fish species including carp [17], [18], tilapia [19], [20], sea bream [21], [22], sea bass [23], [24], rainbow trout [25], salmon [26] and plaice [27]. These transferred maternal molecules have been proposed to be involved in the early defense against pathogens in developing fish embryos and larvae. It has recently been shown that the homogenates of Atlantic salmon embryos have very low hemolytic activities [28]. However, whether the maternal complement components function during early embryogenesis remains uncertain.

In this study, we have sought to establish an *in vitro* system to assay the complement-mediated bacteriolysis in the extracts of fertilized eggs of zebrafish *Danio rerio*, and thereby to examine which complement pathway is responsible for the bacteriolytic activity in the early developing embryos.

## Results

### Bacteriolytic activity of egg cytosol

**Figure 1:**
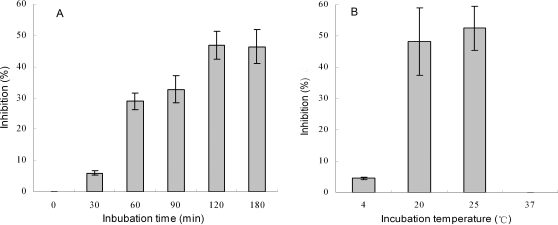
Characteristics of bacteriolytic activity in zebrafish egg cytosol. The egg cytosol filtered through 0.22 µm filter was mixed with *E. coli* suspension and incubated at 25°C for different periods (A) or at different temperature for 2 h (B). The bacteriolytic activities were determined by colony forming unit assay.

The protein concentration of the cytosols prepared from the fertilized eggs of zebrafish *D. rerio* ranged from 13.8 mg/ml to 15.9 mg/ml, with an average of 14.9 mg/ml. The egg cytosol exhibited a conspicuous bacteriolytic activity to *Escherichia coli* in a temperature-dependent manner (Fig. 1A and B). Although the egg cytosol had only slight bacteriolytic activity at low temperature (4°C), the lytic activity increased markedly as temperature rose, with the optimum temperature being 25°C. When the cytosol was pre-incubated at 37°C for 2 h, it lost the bacteriolytic activity completely.

### Complement activity

**Figure 2:**
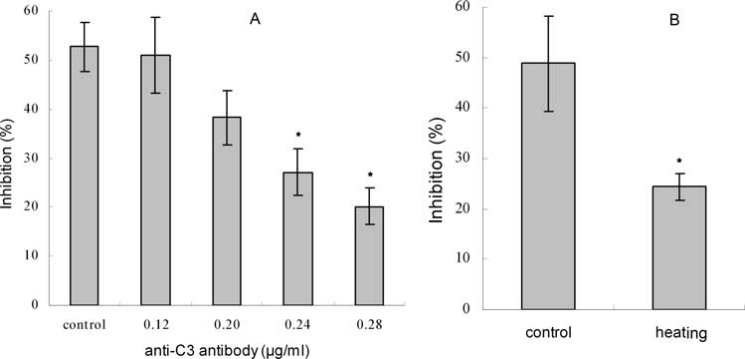
Effects of anti-C3 antibody and heating on the bacteriolytic activity of zebrafish egg cytosol. The egg cytosol was pre-incubated with anti-C3 antibody at different concentrations (A), or inactivated by heating at 45°C (B), and then mixed with *E. coli* suspension. After incubation at 25°C for 2 h, the bacteriolytic activities were measured by colony forming unit assay. * means *p*<0.05.

Pre-incubation of anti-C3 antibody with the egg cytosol was capable of abrogating the bacteriolytic activity to *E. coli* in a concentration-dependent manner (Fig. 2A), which strongly suggested a role for complement in the lytic activity of the egg cytosol. This was further supported by the fact that heating the cytosol (45°C, 30 min) significantly reduced (*p*<0.01) the bacteriolytic activity, with an inhibition rate of 24.3% observed contrasting to that of 48.8% in control (Fig. 2B).

**Figure 3:**
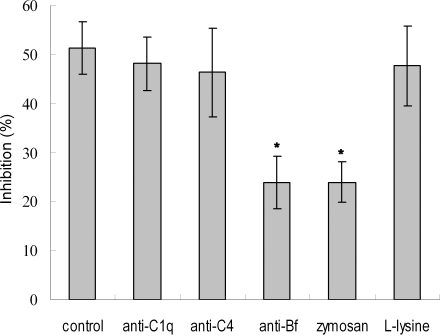
Influences of antibodies and chemical inhibitors on the bateriolytic activity of zebrafish egg cytosol. The egg cytosol was pre-incubated with complement component antibodies and chemicals at optimum concentrations, and mixed with *E. coli* suspension. After incubation at 25°C for 2 h, the bacteriolytic activities were measured by colony forming unit assay.

**Figure 4:**
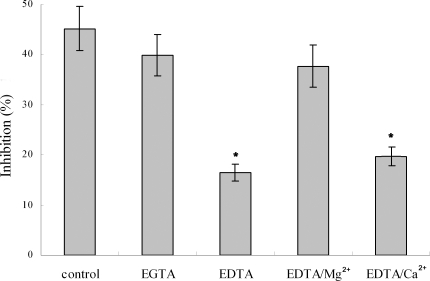
Effects of divalent cation chelators EGTA and EGTA on the bacteriolytic activity of zebrafish egg cytosol. The egg cytosol was pre-incubated with EGTA, EDTA, EDTA with Mg^2+^ or EDTA with Ca^2+^ at optimum concentrations, and then mixed with *E. coli* suspension. After incubation at 25°C for 2 h, the bacteriolytic activities were measured by colony forming unit assay.

It has been shown that C3b can covalently bind to zymosan particles [29], and thus the AP-mediated bacteriolytic activity can be selectively inhibited by addition of zymosan. It was found that the lytic activity of the egg cytosol was significantly decreased by pre-incubation with zymosan, with an inhibition rate of 24.9% contrasting to that of 52.1% in control (Fig. 3). Similarly, pre-incubation with the antibody against Bf, a key enzyme in the AP activation, also resulted in a marked reduction of the lytic activity of the egg cytosol (23.8%; Fig. 3). In contrast, pre-incubation with either mammalian CP inactivator L-lysine [30], [31] or anti-C1q antibody or anti-C4 antibody had little effects on the bacteriolytic activity to *E. coli* (Fig. 3). All these suggested that activation of the AP contributed to the bacteriolytic activity. In agreement, addition of 0.3 mM EGTA to the egg cytosol did not impair the bacteriolytic activity to *E. coli*, while addition of 0.5 mM EDTA to the cytosol significantly depleted its lytic activity to *E. coli* (Fig. 4). Moreover, the bacteriolytic activity of the EDTA-treated egg cytosol was able to be restored by addition of Mg^2+^, but not by addition of Ca^2+^ (Fig. 4).

### Presence of C3 and Bf in egg cytosol

**Figure 5:**
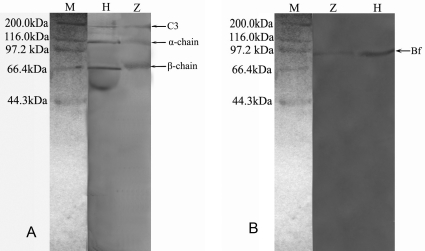
Western blot analysis of C3 and Bf in zebrafish egg cytosol (lane M: molecular marker; lane H: human serum; lane Z: zebrafish egg cytosol). The egg cytosol was electrophoresed on 12% SDS-PAGE gel using the buffer system of Laemmli. The proteins separated were blotted on nitrocellulose membrane and immunostained with rabbit anti-human C3 antibody or goat anti-human Bf antibody, followed by staining with HRP-labeled anti-rabbit IgG and HRP-labeled anti-goat IgG, respectively.

It was shown by Western blotting analyses that both rabbit anti-human C3 and goat anti-human Bf antibodies reacted with human serum and with the egg cytosol. The cytosol was reactive with rabbit anti-human C3 antibody, forming a main band (∼185 kDa) equivalent to C3 and two minor bands (∼115 kDa; ∼70 kDa) resembling C3*α* and C3β chains, respectively (Fig. 5A). Similarly, the egg cytosol reacted with goat anti-human Bf antibody, producing a single positive band of ∼93 kDa, matching that of human Bf (Fig. 5B).

## Discussion

The existence of complement components like C3 and Bf has recently been confirmed in rainbow trout, carp and spotted wolfish. In this study, we found that both C3 and Bf, the key factors functioning in the AP, are also present in the newly fertilized eggs of *D. rerio*, providing the first evidence for a maternal transfer of the complement proteins in zebrafish.

The role of maternal complement conponents in fish eggs has been proposed to be associated with the early defense against pathogens in developing fish embryos and larvae. However, little functional studies have been conducted thus far. Here we showed for the first time that the Gram-negative bacterium *E. coli* is sensitive to lysis by the cytosol prepared from the fertilized eggs of zebrafish *D. rerio*, and all the findings point to complement system being one of the most important factors involved in the bacteriolytic activity observed. First, the bacteriolytic activity was abolished by pre-incubation of anti-C3 antibody with the egg cytosol, a process that would cause the precipitation of the central component of all known complement pathways, C3. Second, the lytic activity was depleted by heating at 45°C, a temperature known to inactivate fish complement [32]. It has been observed that 2-day-old zebrafish embryos possess special macrophages, which have the ability to migrate to sites of infection and engulf bacteria injected [33], [34]. The maternal complement components appear to function in the developing embryos earlier than the embryonic macrophages. It is possible that both macrophages and maternally-derived complement factors can protect the developing zebrafish embryos from microbial attacks.

To determine which pathway of complement activation might be involved in the bacteriolytic activity of the egg cytosol, the antibodies against C1q (a key component of CP), C4 (a key component of both CP and LP) and Bf (a key component of AP) were utilized to block the CP, LP or AP, respectively. It is found that precipitation of C1q and C4 causes little loss of the bacteriolytic activity of the egg cytosol, whereas precipitation of Bf results in a significant reduction of the lytic activity. Furthermore, addition of EGTA to remove Ca^2+^ from the egg cytosol, which can inhibit both CP and LP, induces little decrease in the bacteriolytic activity. In contrast, pre-incubation of EDTA with the egg cytosol leads to a substantial reduction of the bacteriolytic activity, and saturation of the chelator with Mg^2+^ is capable of restoring the lytic activity, but not by addition of Ca^2+^. Moreover, selective inhibition of the AP by zymosan A induces marked loss of bacteriolytic activity, while addition of L-lysine, an inactivator of the CP, is not inhibitory. Taken together, all these undoubtedly indicate that activation of the AP is responsible for the bacteriolytic activity of the egg cytosol, but the CP and LP have little contribution to the lytic activity.

In summary, the complement system operating via the AP plays a crucial role in the developing zebrafish embryos and larvae. As the maternal transfer of immune factors to piscine offspring is widespread, it is highly likely that the complement-mediated killing of pathogens may generally occur in the early developmental stages of fishes. It will be of interest in the future to study if the maternal IgM can aid activation of the AP in the developing fish embryos.

## Materials and Methods

### Reagents

Ethylenediamine tetraacetic acid (EDTA), ethyleneglycol-bis (β-aminoethyl ether)-N,N,N',N'-tetraacetic acid (EGTA), zymosan A, L-lysine and bovine serum albumin (BSA) were purchased from Sigma (USA), and peptone and yeast extract were from OXOID (Japan). Rabbit anti-human C3 antibody was procured from Abcam (UK), and goat anti-human factor B (Bf) antibody from R & D (America). Goat anti-human C1q antibody, Rabbit anti-mouse C4 antibody and horseradish peroxidase (HRP)-labeled rabbit anti-goat IgG were from Boster (China), and HRP-labeled goat anti-rabbit IgG from Zhongshan (China). All other chemicals used were analytical reagents

### Preparation of *Escherichia coli*

The Gram-negative bacterium *E. coli* (P8760) was incubated in LB broth to logarithmic growth phase, and harvested by centrifugation at 3 000 g at 4°C for 10 min. The pellets were washed three times with sterilized 0.9% saline, re-suspended in the saline at a density of 10^6^ cells/ml, and used for the following experiments.

### Preparation of egg cytosol

Mature male and female fishes *D. rerio* were placed in the late evening in a 10 litre tank at a female to male ratio of 2∶1, and maintained at 26±1°C. The naturally fertilized eggs, which were usually at 2- to 8-cell stage, were collected in the next early morning. The unhealthy eggs were removed, and the healthy fertilized eggs were rinsed three times with double-distilled H_2_O and then once with ice-cold double-distilled H_2_O. After the excess H_2_O was withdrawn, the eggs were immediately homogenized on ice for 30 seconds, and centrifuged at 15 000 g at 4°C for 30 min. The supernatant, egg cytosol, was pooled, aliquoted and stored at −70°C until used.

The protein concentrations were determined by the method of Bradford [35] with BSA as standard.

### Assays for bacteriolytic activity

The egg cytosol was filtered through 0.22 µm filter (Millipore) before use. An aliquot of 120 µl of the egg cytosol was mixed with 6 µl of *E. coli* suspension with 10^6^ cells/ml, and the mixture was pre-incubated, with gentle stirring, at 25°C for 2 h. Subsequently, an aliquot of 20 µl of the mixture was sampled at 30, 60, 90, 120 and 180 min, respectively, diluted to a volume of 100 µl with sterilized 0.9% saline, and plated onto 3 LB agar plates (30 µl each plate). After incubation at 37°C for 12 h, the resulting bacterial colonies in each plate were counted. The control was processed similarly except that the cytosol was replaced with sterilized saline. The percent of bacterial growth inhibition by the cytosol was inferred from the difference between the numbers of colonies in the test and control.

To determine the optimum temperature for the bacteriolytic activity, 40 µl of the egg cytosol was mixed with 2 µl of *E. coli* suspension. The mixtures were pre-incubated at 4°C, 20°C, 25°C and 37°C, respectively, for 2 h, and the bacteriolytic activity was then assayed as described above.

### Assays for inhibition of complement

The capacity of the antibodies against C3, Bf, C1q and C4 to inhibit the bacteriolytic activity of the egg cytosol was analyzed by the method of Nonaka et al. [36]. Briefly, 40 µl of the egg cytosol was pre-incubated with anti-C3, C1q, C4 and Bf antibodies at final concentrations of 0.12∼0.28, 0.01, 0.08 and 0.24 µg/ml, respectively, at 25°C for 30 min, followed by addition of 2 µl of *E. coli* suspension. The mixtures were adjusted to a volume of 50 µl with sterilized saline, and incubated at 25°C for 2 h. The bacteriolytic activities were measured as described above. For control, the antibodies were replaced by sterilized saline.

Prior to use, aliquots of 40 µl of the egg cytosol were inactivated by heating at 45°C for 30 min [8], [37], and mixed with 2 µl of *E. coli* suspension. The mixtures were adjusted to 50 µl with sterilized saline, incubated at 25°C for 2 h, and the remaining bacteriolytic activities assayed. The egg cytosol without heating was used in control.

For chelation experiments, aliquots of 40 µl of the egg cytosol were mixed with 1.5 µl of 10 mM EGTA, 2.5 µl of 10 mM EDTA, 2.5 µl of 10 mM EDTA with 2.5 µl of 10 mM MgCl_2_ and 2.5 µl of 10 mM with 2.5 µl of 10 mM CaCl_2_, respectively, and pre-incubated at 25°C for 30 min. The mixtures were then combined with 2 µl of *E. coli* suspension, and adjusted to 50 µl with sterilized saline. After incubatation at 25°C for 2 h, the bacteriolytic activity was determined. Control was processed similarly except that the chelator solutions were replaced by sterilized saline.

To inhibit the classical pathway, 40 µl of the egg cytosol was pre-incubated with 0.6 mM L-lysine at 25°C for 30 min, and then mixed with 2 µl of *E. coli* suspension. The mixtures were adjusted to 50 µl with sterilized saline. After incubation at 25°C for 2 h, the bacteriolytic activity was assayed.

The stock solution of 10 mg/ml zymosan A was prepared by boiling 10 mg zymosan A in 1 ml of 14 mM NaCl for 30 min, followed by centrifugation at 16 000 g for 5 min. To inhibit the alternative pathway, 100 µl (1 mg of zymosan) of the stock solution was centrifuged at 16 000 g for 5 min, and the zymosan pellet was re-suspended in 40 µl of the egg cytosol. After pre-incubation at 25°C for 30 min, the zymosan in the reaction medium was removed by centrifugation at 16 000 g for 5 min. The resulting egg cytosol was mixed with 2 µl of *E. coli* suspension, and adjusted to 50 µl with sterilized saline. The mixture was incubated at 25°C for 2 h, and the bacteriolytic activity was tested.

### Western blotting

To confirm the presence of C3 and Bf in the egg cytosol, Western blotting analysis was carried out. The egg cytosol and human serum were electrophoresed on 12% SDS-polyacrylamide gel electrophoresis (SDS-PAGE) gel using the buffer system of Laemmli [38]. The proteins separated were blotted on nitrocellulose membrane (Hybond, Amersham Pharmacia), and immunostained with rabbit anti-human C3 antibody (1∶1000) and goat anti-human Bf (1∶400) antibody, followed by staining with HRP-labeled anti-rabbit IgG and HRP-labeled anti-goat IgG, respectively [39]. The molecular mass standards used were myosin (200 kDa), β-galactosidase (116 kDa), phosphorylase B (97.2 kDa), serum albumin (66.4 kDa) and ovalbumin (44.3 kDa).

### Statistical analysis

All experiments were performed in triplicate, and repeated at least three times. Data were subjected to statistical evaluation with ANOVA, and difference at *p*<0.05 was considered significant. All data were expressed as a mean±standard deviation (SD).
